# Parental information needs during a large group a streptococcus upsurge: A secondary analysis of a cross-sectional, mixed methods study

**DOI:** 10.1016/j.puhip.2025.100676

**Published:** 2025-10-14

**Authors:** Rebecca Wilson, Louise E. Smith, Maria Theresa Redaniel, Richard Amlôt, Paul R. Hunter, G James Rubin

**Affiliations:** a National Institute for Health and Care Research Applied Research Collaboration West (NIHR ARC West), University Hospitals Bristol and Weston NHS Foundation Trust, UK; b Population Health Sciences, Bristol, Medical School, University of Bristol, UK; c Behavioural Science and Insights Unit, UK Health Security Agency, UK; d National Institute for Health and Care Research Health Protection Research Focus Award (NIHR HPRFA) in Outbreak Related Behaviours, King's College London, UK; e National Cancer Registry Ireland, UK; f Norwich Medical School, University of East Anglia, UK; g Department of Psychological Medicine, King's College London, UK

**Keywords:** Group a streptococcus, Knowledge, Worry, Communication

## Abstract

**Objectives:**

Upsurges of Group A streptococcus (Strep A) and invasive Group A Strep (iGAS) among children can lead to severe health outcomes. Little is known about parents’ information needs during upsurges.

**Study design:**

Secondary analysis of an online cross-sectional survey of 503 parents in the UK, conducted during an upsurge of Strep A/iGAS in 2022.

**Methods:**

Data were available on perceived severity of Strep A and iGAS, levels of worry, knowledge about their symptoms, and whether parents had sought information and if so what.

**Results:**

Thirty-seven participants (7.4 %) had not heard of Strep A, versus 140 (27.8 %) for iGAS. Most participants who had heard of either condition had searched for information, commonly in relation to symptoms. Percentages of participants giving high ratings for severity and worry (scores of 5 or more out of 7) were similar for Strep A (severity: 78.7 %, worry: 65.9 %) and iGAS (severity: 81.1 %, worry: 61.8 %). The only symptoms shown to participants that were correctly identified by more than 50 % were ‘flu-like symptoms’ (recognised by 51.8 % for Strep A and 40.2 % for iGAS) and sore throat (52.5 % and 37.3 % respectively). There was no association between looking for information, or where participants had received information from, and symptom knowledge scores.

**Conclusions:**

High levels of worry existed among parents during the 2022 upsurge in Strep A/iGAS in the UK. This was accompanied by uncertainty about the symptoms associated with Strep A/iGAS and a high desire for more information about symptoms. During future outbreaks, providing clear information about the symptoms to watch out for should continue to be prioritised.

## 1 What this study adds

• During an upsurge of Strep A/iGAS among children, parents must decide when to seek medical attention for their children and when to treat their illness at home.
• During a major upsurge of Strep A/iGAS in the UK in 2022, parents had limited knowledge of the symptoms associated with Strep A/iGAS, reporting high levels of information seeking, and high levels of worry and perceived severity.

## 2 Implications for policy and practice

• During future upsurges of Strep A/iGAS, attention will be needed on providing clear communication to parents with a focus on explaining what symptoms to watch out for.

## 3 Introduction

Group A streptococcus (GAS, also commonly referred to as ‘Strep A’) is a bacterium that can cause a variety of different illnesses. Streptococcal sore throat is the most common presentation, though the bacterium can cause skin infections such as impetigo and erysipelas. Untreated, a simple strep A infection can progress to invasive Group A Strep (iGAS). iGAS occurs when the infection spreads to parts of the body that are normally sterile such as the blood, brain and lungs. Once the Strep A infection becomes invasive it can rapidly progress to death or leave the patient with long term disability [1].

It can often be difficult to distinguish between a streptococcal sore throat which requires antibiotic treatment from the more common viral infections that do not require specific therapy. Similarly it may not be easy to distinguish between a simple strep A infection and iGAS which is a medical emergency needing immediate intervention [2].

In 2022, the UK experienced a widespread Strep A/iGAS upsurge which led to the deaths of at least 45 children [3]. During this period, official guidance advised parents to seek urgent care for their child if they displayed a range of severe symptoms [4]. Evidence at the time suggested that parents had limited knowledge about Strep A/iGAS [5].

In this secondary analysis, we assessed parental information needs using data collected by the UK Health Security Agency (UKHSA) during the 2022 Strep A/iGAS upsurge. We assessed parental information seeking, including topics searched for and if information was found; perceptions of severity and worry relating to Strep A and iGAS; and knowledge of their symptoms. We assessed associations between demographics, information seeking and sources of information, with symptom knowledge.

## 4 Methods

### 4.1 Design

An online cross-sectional survey was conducted by Basis Research (a market research company) on behalf of UKHSA between 19th-21st December 2022. For this analysis, we focused on data from questions asking about information needs, perceptions of severity and worry, and knowledge of symptoms.

### 4.2 Participants

Participants were recruited through an existing online panel (Dynata (https://www.dynata.com/), reach of 70 million people globally). Participants were eligible if they lived in the UK, were 18 years or over, and were a parent or guardian with a child aged under 18 years. The survey used quota sampling based on region, age, gender, and social grade, to ensure participants were broadly representative of parents within the UK. Participants were reimbursed in points which could be redeemed for rewards (∼£0.25 to £0.50).

### 4.3 Data

#### 4.3.1 Socio-demographic factors

UKHSA collected details including age, gender, geographical region, ethnicity, and the age and level of schooling of children. Where participants had multiple children, a mean of their ages was generated. We also generated a binary variable denoting whether participants had at least one child in nursery/primary school/secondary school.

#### 4.3.2 Information seeking

Participants were asked if they had heard of “Strep A/Group A Strep” and “iGAS/Invasive Group A Strep”, “before today”. Those who had heard of either were asked if they had looked for information about them, if they found what they were looking for and what information they were seeking (open-ended question; full items reported in Supplementary Table 1).

Participants reported all sources where they had seen information regarding Strep A or iGAS. A hierarchical variable with one main source for each participant was generated [6], classifying sources as: official, mainstream media, social media, or ‘other’.

#### 4.3.3 Perceived severity, worry and symptom knowledge

Participants rated how serious they perceived Strep A and iGAS to be, on a scale of 1 (‘not at all serious’) to 7 (‘extremely serious’). They rated their worry about their child contracting Strep A or iGAS from 1 (‘not at all worried’) to 7 (‘extremely worried’).

Participants were presented with 12 symptoms, of which nine were symptoms of Strep A and iGAS, including those that were presented on the NHS website for Strep A [4], and three were other common symptoms. They were asked to state whether each symptom was linked to either condition. We calculated variables for each symptom denoting whether the participants had correctly identified it as a symptom/not a symptom of Strep A/iGAS and summed these to create a knowledge score of 0–12. If participants selected ‘all of the above’, they were scored nine. If they selected ‘none of the above’, they were given a score of three. If they responded ‘don't know/not sure’ they were given a score of zero.

### 4.4 Analysis

LES used content analysis to identify and quantify themes within open-ended responses, with GJR checking a sample of codes. Discrepancies were resolved through discussion. Where responses mentioned two themes, both codes were applied.

We used univariate and multivariable ordered logistic regression models to determine the association between parents' age and gender, child's age, whether the parent had a child in nursery/school, ethnicity and region, information source and information seeking, with knowledge of Strep A and iGAS symptoms.

## 5 Results

The survey was completed by 503 parents, with a mean age of 39.1 (standard deviation 9.1). Participants included 233 (46.3 %) men and 270 (53.7 %) women. In terms of ethnicity, 391 were white (77.7 %), 52 Asian (10.3 %), 37 Black (7.4 %), 16 mixed ethnicity (3.2 %) and seven (1.4 %) reported another ethnicity or preferred not to say. Parents came from the North, Scotland or Northern Ireland (n = 166, 33.0 %), the Midlands or Wales (149, 29.6 %), or the South (188, 37.4 %). Four hundred and thirty-seven parents (86.9 %) had at least one child in either nursery or school. The mean age for children was 8.3 (standard deviation 4.9, n = 500). Thirty-seven parents (7.4 %) reported having never heard of Strep A, versus 140 (27.8 %) for iGAS.

### 5.1 Information seeking

Of those who had heard of either Strep A or iGAS (n = 467), most participants had searched for information, finding (n = 190, 40.7 %) or not finding (n = 73, 15.6 %) what they were looking for. Among parents who found the information they needed, the most commonly reported information need related to symptoms (139 out of 240 codes; Supplementary Table 2). Specifically, parents searched for information about signs and symptoms, their duration, early symptoms, how to differentiate between Strep A, iGAS and other illnesses, and for pictures of signs. Example quotes include “symptoms to look out for and when medical advice should be sought”, “pictures of symptoms”, and “symptoms, when to be concerned and what to do if concerned”. Details about symptoms (34 out of 85 codes) were also the most commonly sought-after information for parents who did not find what they were looking for, for example “how to differentiate between normal flu symptoms and invasive Strep A symptoms” and “the symptoms and when to go and not to go to hospital”.

### 5.2 Perceived severity, worry and symptom knowledge

Ratings of perceived severity were similarly high for Strep A and iGAS, with 78.7 % and 81.1 %, respectively, scoring them as five or more out of seven (Table 1). Worry ratings were also similar, with 65.9 % and 61.8 % scoring five or more for Strep A and iGAS respectively.

**Table 1:** Knowledge of, and worry about, Strep A and iGAS. Questions asked to those who had definitely heard of, or thought they had heard of Strep A (n = 461) or iGAS (n = 338).

	Strep A (total n = 461)	iGAS (total n = 338)
How serious do you consider Strep A/iGAS^a^		
1 (‘Not at all serious’) to 3	24 (5.2 %)	15 (4.4 %)
4 (‘Somewhat serious’)	62 (13.4 %)	34 (10.1 %)
5 to 7 (‘Extremely serious’)	363 (78.7 %)	274 (81.1 %)
Don't know/not sure	12 (2.6 %)	15 (4.4 %)
To what extent are you worried about your child contracting Strep A/iGAS^a^		
1 (‘Not at all worried) to 3	64 (13.9 %)	35 (10.4 %)
4 (‘Somewhat worried’)	86 (18.7 %)	80 (23.7 %)
5 to 7 (‘Extremely worried’)	304 (65.9 %)	209 (61.8 %)
Don't know/not sure	7 (1.5 %)	14 (4.1 %)
Correctly identified symptoms of Strep A and iGAS^a^		
Flu-like symptoms	239 (51.8 %)	136 (40.2 %)
Sore throat	242 (52.5 %)	126 (37.3 %)
Rash (raised and sandpapery)	201 (43.6 %)	132 (39.1 %)
Scabs and sores	61 (13.2 %)	61 (18.0 %)
Pain and swelling	88 (19.1 %)	90 (26.6 %)
Severe muscle aches	113 (24.5 %)	115 (34.0 %)
Nausea and vomiting	97 (21.0 %)	77 (22.8 %)
Headache	172 (37.3 %)	106 (31.4 %)
Cough^a^	294 (63.8 %)	237 (70.1 %)
Running nose/sneezing^a^	346 (75.1 %)	272 (80.5 %)
Nose bleeds^a^	436 (94.6 %)	298 (88.2 %)
Fatigue/extreme tiredness	168 (36.4 %)	128 (37.9 %)
All of the above	70 (15.2 %)	74 (21.9 %)
None of the above	3 (0.7 %)	4 (1.2 %)
Don't know/not sure	49 (10.6 %)	47 (13.9 %)

a N (%) respondents that correctly identified these were not symptoms of Strep A/iGAS.

Of genuine Strep A/iGAS symptoms listed in the survey, the most commonly recognised were ‘flu-like symptoms’ (recognised by 51.8 % for Strep A and 40.2 % for iGAS) and sore throat (52.5 % and 37.3 %). The mean (standard deviation) number of symptoms correctly identified was similar for Strep A (5.9 (2.9)) and iGAS (6.1 (3.2)) (Table 1).

In fully adjusted regression models, none of the explanatory variables were associated with knowledge of Strep A or iGAS symptoms (Supplementary Tables 3 and 4).

## 6 Discussion

Whilst most Strep A infections can be managed at home with appropriate antibiotic therapy, iGAS is a medical emergency that requires immediate medical intervention [1]. Thus it is essential that patients or their parents are able to know when to seek emergency care.

During the 2022 Strep A/iGAS upsurge in the UK, most parents had heard of the conditions but had limited knowledge about their symptoms. Only two symptoms were correctly selected by more than half of the participants for Strep A (sore throat and flu-like symptoms), and none of the iGAS symptoms reached even this level of recognition. Symptoms that were commonly misidentified may warrant further investigation using a qualitative approach. Levels of worry and perceptions of severity were equally high for both Strep A and iGAS, likely contributing to the high levels of consultations reported by health services during the period [7].

Active information seeking was common, particularly for information relating to symptoms. Reassuringly, most parents found the information they were looking for, however there was no association between finding information, or source of information, and increased knowledge about symptoms, suggesting that understanding about symptoms remained a particular challenge for parents throughout the upsurge. Other studies conducted during the Strep A outbreak and COVID-19 pandemic have also found an increase in parental information seeking about symptoms [8,9].

The limited understanding, active attempts to find out more and high worry that we identified, indicates a need for detailed information with a particular focus on symptoms and when to seek help for illnesses that are generally mild, but can rapidly become life threatening. This reinforces the need for clear communication about symptoms during outbreaks, including to parents during Strep A/iGAS upsurges.

## Ethical approval

This work was conducted as part of a service evaluation of communications around Strep A/iGAS and so was exempt from requiring ethical approval. Data were processed in line with the Market Research Society code of conduct and consent was implied through participation in the survey (standard practice). When commissioning market research with a supplier, REGG approval is not required, subject to the supplier and the activity meeting certain requirements. Firstly, our suppliers (Basis & Discovery) meet all the requirements set out in the DPS Research & Insights RM6126 Order Form (https://www.crowncommercial.gov.uk/agreements/RM6126), as part of the joining the DPS Framework. They comply with the Market Research Society Code of Conduct, GDPR and meet the relevant ISO standards. The activity does not involve a sensitive topic nor a vulnerable audience.

## Funding

This research was supported by the National Institute for Health and Care Research Applied Research Collaboration West (NIHR ARC West) and the National Institute for Health and Care Research Health Protection Research Focus Award (NIHR HPRFA) in Outbreak Related Behaviours, a partnership between the UK Health Security Agency, King's College London and the University of East Anglia. The views expressed are those of the author(s) and not necessarily those of the NIHR, UKHSA or the Department of Health and Social Care. For the purpose of open access, the author has applied a Creative Commons Attribution (CC BY) licence to any Author Accepted Manuscript version arising.

## Declaration of interests

RW, MTR, and PRH declare no interests. GJR declares payments for research funding, consultancy, speaker fees, advisory board membership and expert witness work relating to Sanofi, AstraZeneca and other life science companies. LES acts as a consultant to the Sanofi group of companies and has supported an expert witness case involving a life sciences company. LES and RA are employed by the UK Health Security Agency.
