# Supplementary material for: Parental information needs during a large group a streptococcus upsurge: A secondary analysis of a cross-sectional, mixed methods study
Source: Public Health Pract (Oxf). 2025 Oct 14;10:100676. doi: 10.1016/j.puhip.2025.100676 (PMC12556354; doi:10.1016/j.puhip.2025.100676)
Supplement: Multimedia component 1 [file mmc1.docx]

## Supplementary Materials

**Supplementary Table 1: Items from UKHSA Strep A questionnaire**

| **Question** | **Response options** |
| --- | --- |
| *Information seeking* | |
| Which of the following have you heard of before today?  *... Strep A / Group A Strep*  *... IGAS / Invasive Group A Strep* | - I’ve definitely heard of this before - I think I've heard of this before - I've never heard of this before - Don’t know / not sure |
| Have you been looking for information on Strep A / Invasive Group A Strep? | - Yes and I have found what I needed - Yes but I haven’t found what I needed - No I haven’t been looking for information - None of the above |
| What information were you looking for? Please give as much detail as possible | Open-ended |
| And where have you seen / heard information regarding Strep A / Invasive Group A Strep (iGAS)?  Please select all that apply | - From official UK Government sources e.g. Gov.uk - From the UK Health Security Agency (UKHSA) - From the NHS - From intergovernmental organisations e.g. the World Health Organisation (WHO), etc. - From my GP (or other local health provider) - In the news e.g. TV, radio, newspapers, etc. - In the news online - Via social media - In international press or TV - From other content shared via social media e.g. from celebrities, influencers, other sources etc. - From online searches e.g. Google, Bing, etc. - From friends / family/ partner - My child’s school or nursery - Other (please specify) - Don’t know / can’t remember - None of the above |
| *Perceived severity, worry, symptom knowledge and confidence in discriminating* | |
| How serious do you consider each of the below infectious diseases to be?  Please answer using the scale below, with 1 being ‘Not at all serious’ and 7 being ‘Extremely serious’  *… Strep A / Group A Strep*  *… iGAS / Invasive Group A Strep* | - 1 – Not at all serious - 2 - 3 - 4 – Somewhat serious - 5 - 6 - 7 – Extremely serious - Don’t know / not sure |
| To what extent are you worried about your child(ren) contracting each of the below infectious diseases?  Please answer using the scale below, with 1 being ‘Not at all worried’ and 7 being ‘Extremely worried’  *… Strep A / Group A Strep*  *… iGAS / Invasive Group A Strep* | - 1 – Not at all worried - 2 - 3 - 4 – Somewhat worried - 5 - 6 - 7 – Extremely worried - Don’t know / not sure |
| Which of the following do you think are symptoms of the below?  Please select all that apply.  *… Strep A / Group A Strep*  *… iGAS / Invasive Group A Strep* | - Flu-like symptoms, such as a high temperature, swollen glands or an aching body - Sore throat - A rash that is raised and feels sandpapery - Scabs and sores - Pain and swelling - Severe muscle aches - Nausea and vomiting - Headache - Cough - Running nose / sneezing - Nose bleeds - Fatigue / extreme tiredness - All of the above - None of the above - Don’t know / not sure |

**Supplementary Table 2: Full results of content analysis of open-ended questions, showing prevalence of information that parents were searching for and found, or searching for and did not find.**

| **Found information** | | | **Did not find information** | | |
| --- | --- | --- | --- | --- | --- |
| **Theme** | N [Total n=240] | Example quote | **Theme** | N [Total n=85] | Example quote |
| **Symptoms** | 139 | “What the symptoms are” | **Symptoms** | 34 | “More detailed information about symptoms” |
| **General information** | 37 | “Knowing more about the disease” | **Transmission** | 15 | “How it is contracted and spread” |
| **What to do if your child is ill** | 34 | “How to treat it”  “…when to seek medical attention if it gets serious” | **General information / details** | 14 | “Has this been around before and how did it come around”  “…Why is it suddenly being talked about? Hasn’t it always been circulating?” |
| **Transmission** | 13 | “How it spreads” | **What to do if your child is ill** | 13 | “How to treat at home” |
| **Severity** | 10 | “How serious the illness is” | **Severity** | 5 | “More about the seriousness of it”  “What is it and how dangerous is it…” |
| **Public health advice / guidance** | 4 | “Isolation rules”  “Guidance” | **Unsure** | 4 | “I am not sure” |
| **Nothing / unsure** | 3 | “Nothing” |  |  |  |

**Supplementary Table 3: Univariate generalised linear regression models (with logit link and binomial family) for Strep A and iGAS symptom knowledge**

|  | Parents’ knowledge of Strep A symptoms^1^,  Coefficient (95% CI) | Parents’ knowledge of iGAS symptoms^2^,  Coefficient (95% CI) |
| --- | --- | --- |
| Parent’s gender (female compared with male) | 0.36 (-0.01 to 0.73) | 0.23 (-0.23 to 0.69) |
| Parent’s age | -0.01 ( -0.03 to 0.01) | <0.01 (-0.03 to 0.03) |
| Child’s age | -0.005 (-0.04 to 0.03) | 0.01 (-0.04 to 0.07) |
| Had at least one child in nursery/school (compared with not having any children in nursery/school) | 0.21 (-0.34 to 0.75) | 0.26 (-0.43 to 0.94) |
| Ethnicity (compared with white) |  |  |
| Asian | 0.03 (-0.60 to 0.66) | -0.19 (-0.98 to 0.61) |
| Black | -0.03 (-0.75 to 0.69) | -0.02 (-0.86 to 0.82) |
| Mixed | 0.14 (-0.89 to 1.18) | 0.11 (-1.12 to 1.35) |
| Other | 0.03 (-1.73 to 1.80) | -0.30 (-2.74 to 2.14) |
| Region (compared to South) |  |  |
| Midlands, Wales | 0.03 (-0.42 to 0.48) | 0.10 (-0.44 to 0.64) |
| North, Scotland, NI | -0.03 (-0.47 to 0.41) | <0.01 (-0.53 to 0.55) |
| Information source (compared with official sources) |  |  |
| Mainstream media | ***-0.44 (-0.84 to -0.05)*** | -0.30 (-0.81 to 0.20) |
| Social media | -0.28 (-0.95 to 0.39) | -0.03 (-0.88 to 0.82) |
| None of the above | -0.82 (-1.73 to 0.10) | -0.60 (-1.71 to 0.50) |
| Information seeking and perceived sufficiency of information (compared with ‘No, I haven’t been looking for information’) |  |  |
| Yes and I have found what I needed | 0.40 (>-0.01 to 0.80) | 0.31 (-0.21 to 0.82) |
| Yes but I haven’t found what I needed | 0.27 (-0.27 to 0.81) | 0.14 (-0.52 to 0.81) |
| None of the above | -0.31 (-1.87 to 1.25) | -0.46 (-2.28 to 1.37) |

^1^Includes parents who had definitely heard of, or thought they had heard of, Strep A (N=461), except for Child’s age, which had missing data and included N=459 parents

^2^Includes parents who had definitely heard of, or thought they had heard of, iGAS (N=338), except for Child’s age, which had missing data and included N=336 parent

**Supplementary Table 4: Multivariable generalised linear regression models for Strep A and iGAS symptom knowledge (with logit link and binomial family)**

|  | **Parents’ knowledge of Strep A symptoms^1^,**  **Coef (95% CI)** | **Parents’ knowledge of iGAS symptoms^2^,**  **Coef (95% CI)** |
| --- | --- | --- |
| **Parent’s gender (female compared with male)** | 0.36 (-0.04 to 0.76) | 0.23 (-0.23 to 0.69) |
| **Parent’s age** | <0.01 (-0.02 to 0.03) | <0.01 (-0.03 to 0.03) |
| **Child’s age** | 0.01 (-0.04 to 0.06) | 0.01 (-0.04 to 0.07) |
| **Had at least one child in nursery/school (compared with not having any children in nursery/school)** | 0.19 (-0.40 to 0.78) | 0.26 (-0.43 to 0.94) |
| **Ethnicity (compared with white)** |  |  |
| Asian | -0.09 (-0.74 to 0.57) | -0.19 (-0.98 to 0.61) |
| Black | -0.08 (-0.83 to 0.68) | -0.02 (-0.86 to 0.82) |
| Mixed | 0.16 (-0.90 to 1.21) | 0.11 (-1.12 to 1.35) |
| Other | 0.03 (-1.82 to 1.89) | -0.30 (-2.74 to 2.14) |
| **Region (compared to South)** |  |  |
| Midlands, Wales | 0.06 (-0.40 to 0.52) | 0.10 (-0.44 to 0.64) |
| North, Scotland, NI | 0.01 (-0.45 to 0.47) | <0.01 (-0.53 to 0.55) |
| **Information source (compared with official sources)** |  |  |
| Mainstream media | -0.32 (-0.75 to 0.12) | -0.30 (-0.81 to 0.20) |
| Social media | -0.26 (-0.95 to 0.43) | -0.03 (-0.88 to 0.82) |
| None of the above | -0.69 (-1.64 to 0.26) | -0.60 (-1.71 to 0.50) |
| **Information seeking and perceived sufficiency of information (compared with ‘No, I haven’t been looking for information’)** |  |  |
| Yes and I have found what I needed | 0.26 (-0.18 to 0.71) | 0.31 (-0.21 to 0.82) |
| Yes but I haven’t found what I needed | 0.20 (-0.37 to 0.77) | 0.14 (-0.52 to 0.81) |
| None of the above | -0.18 (-1.78 to 1.42) | -0.46 (-2.28 to 1.37) |

^1^Includes those who definitely, or who thought they had heard of Strep A, with complete data for all variables (N=459)

^2^Includes those who definitely, or who thought they had heard of iGAS, with complete data for all variables (N=336)

^3^Includes those who definitely, or thought they had heard of both Strep A and iGAS, with complete data for all variables (N=317)
